# LPS promotes resistance to TRAIL-induced apoptosis in pancreatic cancer

**DOI:** 10.1186/s13027-017-0139-4

**Published:** 2017-05-30

**Authors:** Katharina Beyer, Lars Ivo Partecke, Felicitas Roetz, Herbert Fluhr, Frank Ulrich Weiss, Claus-Dieter Heidecke, Wolfram von Bernstorff

**Affiliations:** 1 Department of General, Visceral, Thoracic and Vascular Surgery, Universitätsmedizin Greifswald, Greifswald, Germany; 2 Department of General, Visceral and Vascular Surgery, Charité Universitätsmedizin Berlin, Campus Benjamin Franklin, Hindenburgdamm 30, 12203 Berlin, Germany; 3 Department of Obstetrics and Gynaecology, Universitätsklinikum Heidelberg, Heidelberg, Germany; 4 Department of Medicine A, Universitätsmedizin Greifswald, Greifswald, Germany

**Keywords:** TRAIL, LPS, Pancreas cancer, Apoptosis, Cell lines

## Abstract

**Background:**

Though TRAIL has been hailed as a promising drug for tumour treatment, it has been observed that many tumour cells have developed escape mechanisms against TRAIL-induced apoptosis. As a receptor of LPS, TLR 4, which is expressed on a variety of cancer cells, can be associated with TRAIL-resistance of tumour cells and tumour progression as well as with the generation of an anti-tumour immune response.

**Methods:**

In this study, the sensitivity to TRAIL-induced apoptosis as well as the influence of LPS-co-stimulation on the cell viability of the pancreatic cancer cell lines PANC-1, BxPC-3 and COLO 357 was examined by FACS analyses and a cell viability assay. Subsequently, the expression of TRAIL-receptors was detected via FACS analyses. Levels of osteoprotegerin (OPG) were also determined using an enzyme-linked immunosorbent assay.

**Results:**

PANC-1 cells were shown to be resistant to TRAIL-induced apoptosis. This was accompanied by significantly increased osteoprotegerin levels and a significantly decreased expression of DR4.

In contrast, TRAIL significantly induced apoptosis in COLO 357 cells and to a lesser degree in BxPC-3 cells. Co-stimulation of COLO 357 as well as BxPC-3 cells combining TRAIL and LPS resulted in a significant decrease in TRAIL-induced apoptosis. In COLO 357 cells TRAIL-stimulation decreased the levels of OPG thereby not altering the expression of the TRAIL-receptors 1–4 resulting in a high susceptibility to TRAIL-induced apoptosis. Co-stimulation with LPS and TRAIL completely reversed the effect of TRAIL on OPG levels reaching a 2-fold increase beyond the level of non-stimulated cells resulting in a lower susceptibility to apoptosis.

In BxPC-3, TRAIL stimulation decreased the expression of DR4 and significantly increased the decoy receptors TRAIL-R3 and TRAIL-R4 leading to a decrease in TRAIL-induced apoptosis. OPG levels remained unchanged. Co-stimulation with TRAIL and LPS further enhanced the changes in TRAIL-receptor-expression promoting apoptosis resistance.

**Conclusions:**

Here it has been shown that TRAIL-resistance in pancreatic cancer cells can be mediated by the inflammatory molecule LPS as well as by different expression patterns of functional and non-functional TRAIL-receptors.

## Background

Pancreatic cancer remains a devastating disease which displays resistance to even the most aggressive treatment regime [1]. There is increasing evidence that the development and progression of exocrine pancreatic cancer can be promoted by chronic inflammation [2–4]. This connection of inflammation with tumour progression can be mediated by components of the bacterial cell wall like lipopolysaccharide (LPS) from gram-negative bacteria [5]. LPS interacts with immune cells of the tumour microenvironment which, in turn, is especially important in tumour development and progression [2, 6]. Additionally, LPS can directly interact with pancreatic cancer cells increasing the invasive ability of these cells [5]. LPS is recognized by the Toll-like receptor (TLR) 4. TLRs are a family of pattern recognition receptors exerting an important role in host defence against infections [7]. Apart from the expression of TLR4 by cells of the immune system, TLRs have been linked to several cancers including pancreatic cancer [5, 8–14]. In these tumours, LPS can lead to activation of NFkB thus promoting cancer progression and chemoresistance [13].

TNF-related apoptosis inducing ligand (TRAIL) is involved in tumour surveillance. It induces apoptosis upon binding to its receptors death receptor (DR) 4 (TRAIL-receptor 1) and DR5 (TRAIL-receptor 2). Additionally, there are three more TRAIL receptors lacking a functionally active death domain: TRAIL-receptor (TRAIL-R) 3 (decoy receptor 1 or DcR1), TRAIL-R 4 (decoy receptor 2 or DcR2) and the soluble receptor osteoprotegerin (OPG). Thus, they are unable to transmit apoptosis-inducing signals [15]. Several factors determine whether a cell becomes apoptotic following TRAIL-binding: Firstly, many cells express these decoy-receptors inhibiting TRAIL at the membrane level: TRAIL-R3 (DcR1) competes for TRAIL binding, sequestering TRAIL in lipid rafts. TRAIL-R4 (DcR2) inhibits activation of caspase 8 through the formation of heteromeric complexes [16]. Secondly, the cell cycle progression can change the balance of pro- and anti-apoptotic proteins. These proteins collectively help to regulate the signal generated by binding of TRAIL to DR4 or DR5. Thus, a preponderance of anti-apoptotic proteins may result in TRAIL-resistance [17]. Especially members of the Bcl-2 family, cFLIP and IAPs (inhibitor of apoptosis proteins) contribute to TRAIL receptor-signal-transduction pathways leading to TRAIL resistance [17–19].

Despite TRAIL-R1/2 – expression, most pancreatic cancer cells show resistance to TRAIL-induced apoptosis [20–23] mediated by a high activation level of NF-kappaB [24] which enhances the expression of inhibitors of apoptosis like XIAP and FLIP [20–23].

In human colon cancer cell lines, it has been detected by Tang et al. [25] that LPS binding to TLR-4 did not affect the expression of TLR4 nor proliferation of respective cell lines. However, LPS activated NFkB thereby inducing resistance to TRAIL-induced apoptosis [25].

For human lung cancer cell lines, it has been shown that TLR 4 ligation by LPS led to production of anti-inflammatory cytokines as well as resistance of human lung cancer cells to TNFa- and TRAIL-mediated apoptosis. Furthermore, binding of LPS to its receptor TLR4 can activate NFkB thereby promoting resistance to TRAIL – induced apoptosis [26].

In the present study, the effects of LPS stimulation on TRAIL-induced apoptosis in several pancreatic cancer cell lines were examined.

## Methods

### Cell lines

The human pancreatic cancer cell lines PANC-1 and BxPC-3 were obtained from the ATCC *(American Type Culture Collection, Manassas, VA, USA)*. The cell line COLO 357 was obtained from ECACC (European Collection of Authenticated Cell Cultures, London, UK). Cells were maintained in RPMI 1640 (Gibco cell culture, Karlsruhe, Germany) supplemented with 10% heat-inactivated fetal bovine serum (FCS) (PAA, Pasching, Austria) and 1% penicilline/streptomycine (Gibco cell culture) at 37 °C in a 5% CO2 atmosphere at 85% humidity.

### LPS and TRAIL stimulation

Human recombinant TRAIL (purity 95%, endotoxin level < 1.0 EU per 1 g protein) was purchased from Biomol (Hamburg, Germany) and dissolved in RPMI medium. Lipopolysaccharide (E. coli) was purchased from Sigma Aldrich (Munich, Germany). Cells were seeded in six well plates. After 12 h, respective amounts of TRAIL (100 ng/ml or 300 ng/ml) and/or LPS (1 μg/ml) were added and cells were stimulated for 24 h.

### Detection of apoptotic cells

Apoptotic cell death was assessed using an Annexin V Apoptosis Detection Kit (BD Bioscience, Heidelberg, Germany) according to manufacturer’s instructions. Annexin V-positive cells were detected by flow cytometry (FACS Canto, Becton Dickinson, Heidelberg, Germany).

To confirm the results, a propidium iodide cell cycle analysis was performed. For this assay, cells were harvested, stored on ice and washed three times with 2% FCS in PBS. A total of 10^5^ cells were fixed in 70% ethanol and incubated with 0.025 M sodium citrate and 0.067 M disodium phosphate. The pellets were washed with PBS plus 5% FCS, resuspended in 30 μl RNase (1 mg/mL; Qiagen, Hilden, Germany) and stained with 25 μg/mL propidium iodide (Sigma Aldrich). Apoptosis was measured by flow cytometry (FACS Canto, Becton Dickinson, Heidelberg, Germany) employing a standard protocol as described before [27]. In detail, cells were stored on ice and washed 3 times with 2% fetal calf serum (Biochrom) in PBS. A total of 10^5^ cells were fixed in 70% ethanol and incubated with 0.025 M sodium citrate and 0.067 M disodium phosphate at pH 7.8 at room temperature. The pellets were washed with PBS plus 5% FCS, resuspended in 30 μL RNase (1 mg/mL; Qiagen, Hilden, Germany) and stained with 25 g/mL propidium iodide (Sigma Aldrich, Munich, Germany). Apoptosis was measured by FlowCytometry (FACS Canto, Becton Dickinson). Debris were gated out in the FSC versus SSC plot. Singlets were manually gated in the FL2A versus FL2W plot. The hypodiploid DNA peaks in singlevariable DNA histograms were identified. Data were analysed using BD CellQuest Pro.

### Cell viability assay

A Cell Titer Blue Assay (Promega, Mannheim, Germany) was performed according to the manufacturer’s instructions. In brief, Cell Titer Blue substrates were added and plates were incubated for 4 h at 37 °C. Subsequently, fluorescence (excitation at 544 nm and emission at 590 nm) was measured on a plate reader. Triplicates were run for each measurement and means were calculated. Controls without cells were run in parallel.

### FACS analyses of TRAIL-receptors

After stimulation cells were harvested using accutase (Sigma Aldrich) and washed with PBS. After blocking with FcBlock (BD Bioscience, Heidelberg, Germany) cells were labelled with appropriate antibodies according to the manufacturer’s instructions. Purified mouse monoclonal antibodies of anti-human DR4, anti-human DR5, anti-human DcR1 and anti-human DcR2 as well as appropriate secondary antibodies and isotype controls were purchased from Pierce (ThermoFisher Scientific, Schwerte, Germany). FACS analyses were performed using the above flow cytometer. FACS data were analysed using WinMDI.

### Osteoprotegerin ELISA

Levels of osteoprotegerin within the cell culture supernatants were detected using an osteoprotegerin – ELISA (Bender MedSystems, Vienna, Austria) according to the manufacturer’s recommondations.

### Real time quantitative PCR

After stimulation of respective cell lines, cells were harvested and washed with PBS. RNA was extracted using an RNeasy kit (Qiagen, Hilden, Germany) and quantified by spectrophotometry. Subsequently, cDNA was prepared using an RT2 PCR Array first strand kit (Qiagen). Respective primers (i.e. TLR4, GAPDH and becta-actin) (Qiagen) were used according to the manufacturer’s recommondations. Realtime PCR amplification was conducted using an ABI Prism 7000 (ThermoFisher Scientific). Data were analysed using a SuperArray PCR Data analyses software using the comparative threshold method. Data were normalized to the house keeping genes GAPDH and beta-actin.

### Statistics

Results were statistically analysed using the program Graph Pad Prism for Macintosh. The Mann Whitney *U* test was employed to compare means of values of experiments in order to test two independent samples. An ANOVA test was employed to compare more than two independent samples. A p-value below 0.05 was considered to be statistically significant. All data are expressed as mean standard error of the mean.

## Results

### TRAIL – stimulation decreased viability of COLO 357 and BxPC-3 whereas viability of PANC-1 cells remained almost unchanged

**Fig. 1:**
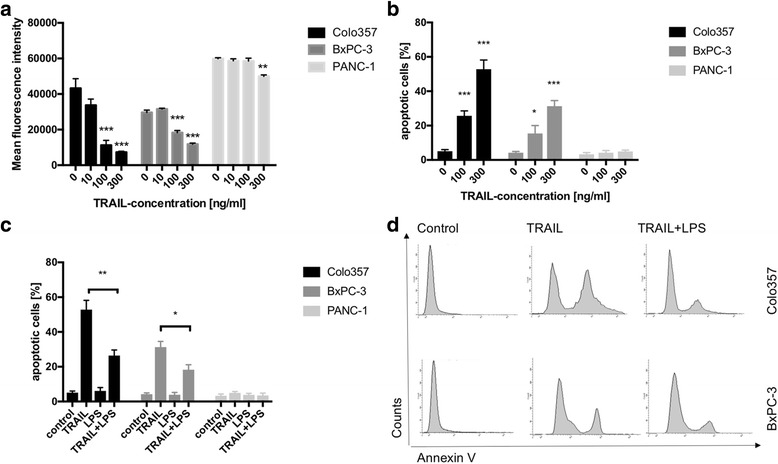
LPS-stimulation promoted resistance to TRAIL-induced apoptosis. **a** Cells cultures of COLO357, BxPC-3 and PANC-1 were stimulated with TRAIL for 24 h and cell viability was determined using a Cell titer blue assay. Mean fluorescence intensities following TRAIL-stimulation are shown. TRAIL – stimulation decreased viability of COLO 357 and BxPC-3 whereas viability of PANC-1 cells remained almost unchanged. *N* = 5/group. **b** Cell cultures of COLO357, BxPC-3 and PANC-1 were TRAIL-stimulated for 24 h and the fraction of apoptotic cells was determined via FACS analyses employing a Annexin V assay. TRAIL-stimulation induced apoptosis in COLO 357 and, to a lesser degree, in BxPC-3 cells, whereas PANC-1 cells were TRAIL-resistant. *N* = 5/group. **c** Cell cultures of COLO357, BxPC-3 and PANC-1 were stimulated with 300 ng/ml TRAIL, 1 μg/ml LPS and 300 ng/ml TRAIL + 1 μg/ml LPS for 24 h. Non-stimulated cell cultures served for controls. Thereafter, fractions of apoptotic cells were determined. Co-stimulation with TRAIL and LPS significantly decreased the number of TRAIL-induced apoptotic cells in COLO357 and BxPC-3. *N* = 5/group. **d** Representative histograms of FACS analyses employing the Annexin V assay of COLO357 and BxPC-3 are shown. Means and standard errors of the mean are shown. *) *p* < 0.05; **) *p* < 0.01; ***) *p* < 0.001

To determine the impact of TRAIL-stimulation on cell viability, cell cultures of PANC-1, BxPC-3 and COLO 357 were TRAIL-stimulated for 24 h and cell viability was determined by a CellTiter Blue Assay. In COLO 357 cells, the mean fluorescence intensity (MFI) ratio was 43256 ± 5347 without stimulation. TRAIL-stimulation decreased the MFI ratio in a dose-dependent manner (10 ng/ml: 33701 ± 3486 (*p* = 0.17, *n* = 5); 100 ng/ml 11213 ± 2784 (*p* = 0.0007 when compared to non-stimulated control; *p* = 0.001 when compared to 10 ng/ml TRAIL; *n* = 5); 300 ng/ml 7276 ± 589 (*p* = 0.0002 when compared to non-stimulated control, *p* = 0.0001 when compared to 10 ng/ml TRAIL, *p* = 0.01 when compared to 100 ng/ml TRAIL; *n* = 5; Fig. 1a).

In BxPC-3 cell cultures, stimulation with 10 ng/ml TRAIL did not significantly alter the cell viability (MFI ratio 29644 ± 1356 versus 31537 ± 479, *p* = 0.22; *n* = 5; Fig. 1a). However, higher concentrations of TRAIL led to decreased amounts of viable cells (100 ng/ml TRAIL: 18292 ± 1189 (*p* = 0.0002 when compared to non-stimulated control, *p* = 0.0001 when compared to 10 ng/ml TRAIL); 300 ng/ml TRAIL: 11853 ± 589 (*p* = 0.0001 when compared to non-stimulated control, *p* = 0.0001 when compared to 10 ng/ml TRAIL, *p* = 0.0012 when compared to 100 ng/ml TRAIL); *n* = 5; Fig. 1a). The pancreatic cancer cell line PANC-1 was the most resistant pancreatic cancer cell line of those tested. TRAIL-stimulation with at least 300 ng/ml did significantly reduce cell viability but only to a minor degree (without stimulation: 59872 ± 548; 10 ng/ml TRAIL: 58714 ± 1125; 100 ng/ml TRAIL: 58562 ± 1593; 300 ng/ml TRAIL: 49993 ± 783 (*p* = 0.001 when compared to 300 ng/ml TRAIL; *n* = 5) (Fig. 1a).

### TRAIL-stimulation induced apoptosis in COLO 357 and, to a lesser degree, in BxPC-3 cells, whereas PANC-1 cells were TRAIL-resistant

The amounts of apoptotic cells induced by TRAIL-stimulation were detected by an Annexin V assay.

Whereas the fraction of apoptotic cells was only 5.1 ± 0.94% in non-stimulated cell cultures of COLO 357, TRAIL-stimulation for 24 h led to a dose-dependent induction of apoptosis (25.7 ± 2.89% in COLO357 cell cultures stimulated with 100 ng/ml TRAIL (*p* = 0.0001) and 52.8 ± 5.39% following stimulation with 300 ng/ml TRAIL (*p* < 0.0001 when compared to non-stimulated control, *p* = 0.0022 when compared to 100 ng/ml TRAIL; *n* = 5, Fig. 1b).

When examining cell cultures of BxPC-3, TRAIL-stimulation with 100 ng/ml significantly enhanced the fraction of apoptotic cells (4.2 ± 0.78% versus 15.43 ± 4.58%, *p* = 0.042; *n* = 5; Fig. 1b). This effect was further increased when stimulating with 300 ng/ml TRAIL reaching 31.32 ± 3.27% apoptotic cells (*p* < 0.0001 when compared to non-stimulated cells, *p* = 0.0224 when compared to 100 ng/ml TRAIL, *n* = 5).

In marked contrast, in PANC-1 cell cultures the fraction of apoptotic cells remained below 5% following TRAIL stimulation indicating that PANC-1 is a cell line resistant to TRAIL-induced apoptosis.

These results indicate that COLO 357 displays a high susceptibility towards TRAIL-induced apoptosis whereas PANC-1 cell cultures are TRAIL-resistant. Cultures of BxPC-3 cells display intermediate sensibilities against TRAIL-induced apoptosis.

These data were confirmed using a propidium iodide cell cycle assay (data not shown).

### LPS stimulation inhibited TRAIL-induced apoptosis

To assess an effect of LPS stimulation on apoptosis induction by TRAIL, respective cell cultures were stimulated with 300 ng/ml TRAIL, 1 μg/ml LPS and 300 ng/ml TRAIL + 1 μg/ml LPS. Thereafter, fractions of apoptotic cells were determined. In the TRAIL-resistant pancreatic cancer cell line PANC-1, co-stimulation with LPS and TRAIL did not lead to any significant alterations in the fraction of apoptotic cells. Thus, there were 3.28 ± 1.0% apoptotic cells without stimulation. LPS-stimulation revealed 3.8 ± 0.9% apoptotic cells. Stimulation with 300 ng/ml TRAIL and LPS resulted in 3.5 ± 1.3% apoptotic cells.

In non-stimulated COLO 357 cells, there were 5.1 ± 0.94% cells apoptotic. TRAIL-stimulation led to 52.8 ± 5.39% apoptotic cells (*p* < 0.0001, *n* = 5) whereas LPS-stimulation revealed 6.1% ± 1.90% apoptotic cells (*n* = 5, *p* = 0.66 when compared to non-stimulated cells, Fig. 1c). Co-stimulation with TRAIL and LPS partially reversed the effect of TRAIL on apoptosis-induction revealing 26.4 ± 3.21% apoptotic cells (*n* = 5, *p* = 0.003 when compared to TRAIL-stimulated cell cultures; Fig. 1c).

In BxPC-3 cultures, there were 4.2 ± 0.78% apoptotic cells. TRAIL treatment led to 31.32 ± 3.27% apoptotic cells (*n* = 5, *p* < 0.0001). Co-stimulation with TRAIL and LPS significantly decreased the number of apoptotic cells reaching 18.3 ± 2.84% (*n* = 5, *p* = 0.0169 when compared to TRAIL-stimulated cell cultures) whereas stimulation with LPS alone did not have an impact on apoptosis (3.9 ± 1.3%, Fig. 1c).

### In PANC-1 cells TRAIL-stimulation decreased the expression of DR4, DR5 and DcR2 as well as increasing the expression of osteoprotegerin (OPG)

**Fig. 2:**
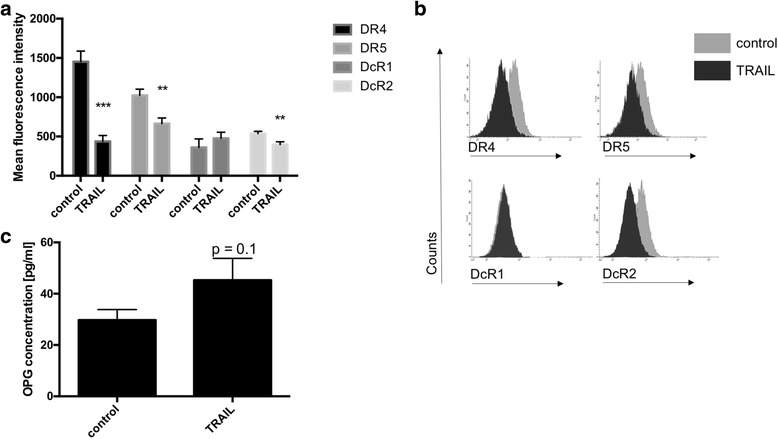
TRAIL-stimulation decreased the expression of DR4, DR5 and DcR2 as well as increasing the expression of osteoprotegerin (OPG) in PANC-1 cells. Cultures of PANC-1 cells were stimulated with 300 ng/ml TRAIL for 24 h. The expression of the TRAIL-receptors 1–4 was detected by FACS analyses. The concentrations of OPG within the supernatants were determined by ELISA. **a** The expression of respective TRAIL-receptors is expressed as mean fluorescence intensity. TRAIL-stimulation of PANC-1 cells led to significantly decreased expressions of DR4 (TRAIL receptor 1), DR5 (TRAIL receptor 2) and DcR2 (TRAIL receptor 4) whereas the expression of DcR1 (TRAIL receptor 3) remained unaltered. N ≥ 6/group. **b** Representative histograms show the expression of TRAIL-receptors 1 – 4 in TRAIL-stimulated PANC-1 cells and unstimulated controls. **c** The concentration of OPG within the cell culture supernatant increased following TRAIL-stimulation. *N* = 7/group. Means and standard errors of the mean are shown. *) *p* < 0.05; **) *p* < 0.01; ***) *p* < 0.001

As shown by FACS analyses, PANC-1 cells expressed the TRAIL-receptors DR4, DR5, DcR1 and DcR2 (Fig. 2a,b). Additionally, significant amounts of OPG were detected in the supernatants of PANC-1 cell cultures (Fig. 2c). Following TRAIL-stimulation, the expression of the death receptors DR4 and DR5 by PANC-1 cells was decreased. For DR4 the mean fluorescence intensity (MFI) was 1397 ± 145.8 for non-stimulated cell cultures, whereas TRAIL-stimulated cell cultures showed a mean fluorescence intensity of 436.8 ± 76.0 (*p* = 0.0001, *n* = 6/group). For DR5 the mean fluorescence intensity was 1025 ± 77.8 in non-stimulated cells, whereas TRAIL-stimulation led to a mean fluorescence intensity of 663.6 ± 72.5 (*p* = 0.005, n ≥ 7/group). The expression of DcR1 remained unchanged following TRAIL-stimulation (MFI 361.4 ± 108.4, *n* = 5 versus 477.0 ± 77.5, *n* = 7, *p* = 0.39). In contrast, the expression of DcR2 significantly decreased following TRAIL-treatment. The MFI of DcR2 was 537.5 ± 28.9 in non-stimulated cell cultures but decreased to 396.8 ± 25.3 following TRAIL-stimulation (*p* = 0.009; Fig. 2a).

The levels of OPG within the cell culture supernatants of PANC-1 cell cultures were 29.71 ± 4.1 pg/ml in non-stimulated controls. TRAIL-stimulation increased the concentration of OPG within the supernatants revealing 45.29 ± 8.5 (*n* = 7, *p* = 0.1).

### LPS stimulation significantly affected the impact of TRAIL on TRAIL-receptor-expression

**Fig. 3:**
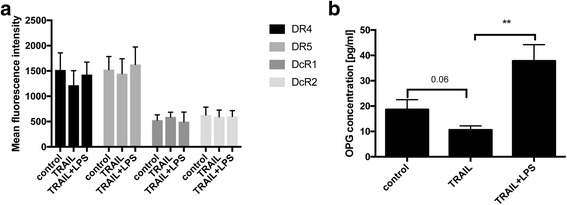
Co-stimulation of COLO357 cultures with LPS and TRAIL increased OPG-levels. Cultures of COLO357 were stimulated with 300 ng/ml TRAIL, 1 μg/ml LPS and 300 ng/ml TRAIL + 1 μg/ml LPS for 24 h. Non-stimulated cell cultures served as controls. The concentration of OPG within the supernatants of respective cell cultures was measured by ELISA. The expression of the TRAIL-receptors 1–4 was determined via FACS analysis. **a** The expression of respective TRAIL-receptors was determined by FACS analysis. Data are expressed as mean fluorescence intensity. *N* = 5/group. **b** Concentrations of OPG within the supernatants of COLO357 cultures are depicted. Whereas TRAIL-stimulated cultures displayed a trend toward decreased OPG-levels, co-stimulation with LPS and TRAIL increased OPG-levels when compared to non-stimulated controls. *N* = 6/group. Means and standard errors of the means are shown. **) *p* < 0.01

As shown by FACS analyses, COLO 357 cells expressed the TRAIL-receptors 1–4. Neither TRAIL-stimulation alone nor LPS-stimulation alone significantly altered the expression of TRAIL-receptors by COLO 357 cells (Fig. 3a). In contrast, TRAIL-stimulation significantly decreased the levels of OPG: Whereas the concentration of OPG in the cell culture supernatants was 18.67 ± 3.8 pg/ml in non-simulated cell cultures, TRAIL stimulation decreased the OPG secretion reaching 10.60 ± 1.5 pg/ml (*p* = 0.06). This effect was significantly reversed by co-stimulation with LPS: Co-stimulation with TRAIL and LPS increased the OPG concentration reaching 37.83 ± 6.4 pg/ml (*p* = 0.0044 when compared to TRAIL-stimulated COLO 357 cultures, *n* = 6/group, Fig. 3b).

**Fig. 4:**
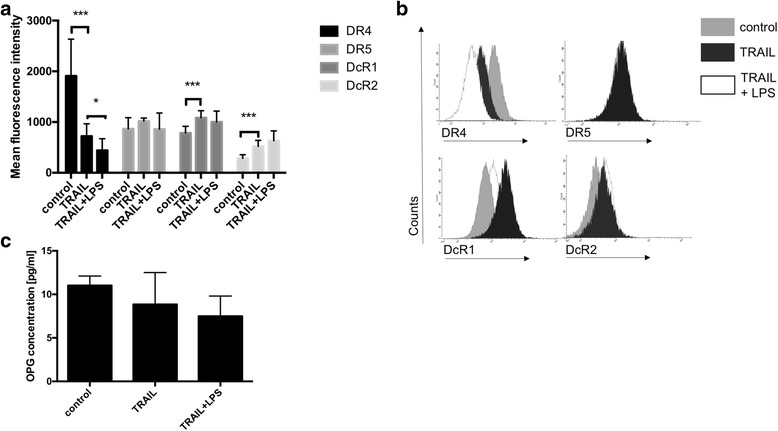
Co-stimulation with TRAIL and LPS significantly affected the impact of TRAIL on TRAIL-receptor-expression in BxPC-3 cells. Cultures of BxPC-3 were stimulated with 300 ng/ml TRAIL, 1 μg/ml LPS and 300 ng/ml TRAIL + 1 μg/ml LPS for 24 h. Non-stimulated cell cultures served as controls. The concentration of OPG within the supernatants of respective cell cultures was measured by ELISA. The expression of the TRAIL-receptors 1–4 was determined via FACS analysis. **a** The expression of the TRAIL-receptors 1–4 by BxPC-3 cells was measured by FACS analysis and is depicted as mean fluorescence intensity. TRAIL-stimulation highly significantly decreased the expression of TRAIL-receptor 1 (DR4) on BxPC-3 cells. N ≥ 6/group. **b** Representative histograms are shown. **c** Concentrations of OPG within the supernatants of BxPC-3 cultures are depicted. Neither stimulation with TRAIL alone nor co-stimulation with TRAIL and LPS significantly altered the levels of OPG. *N* = 8/group. Means and standard errors of the mean are shown. *) *p* < 0.05; **) *p* < 0.01; ***) *p* < 0.001

In contrast, in BxPC-3 cells neither stimulation with TRAIL alone nor co-stimulation with TRAIL and LPS significantly altered the levels of OPG: The concentration of OPG within supernatants of BxPC-3 cultures was 11.0 ± 1.1 pg/ml without stimulation and 8.9 ± 1.3 pg/ml following TRAIL-stimulation (*n* = 8/group, *p* = 0.66 when compared to non-stimulated cell cultures). After co-stimulation with TRAIL and LPS, the concentration of OPG was determined as 7.5 ± 0.8 pg/ml (*n* = 8/group, *p* = 0.25 when compared to TRAIL-stimulated cell cultures, Fig. 4b).

However, TRAIL stimulation significantly decreased the expression of DR4 whereas the expression of DR5 was increased albeit not to a significant degree (DR4: 1908 ± 296.6 for non-stimulated cell cultures versus 722.4 ± 86.0 for TRAIL-stimulated cell cultures, *n* = 6, *p* = 0.0009; DR5: 866.2 ± 77.6 versus 1018 ± 22.0, *p* = 0.08). In contrast, the expression of the decoy receptors DcR1 and DcR2 was increased in TRAIL-stimulated BxPC-3 cell cultures. The mean fluorescence intensity for DcR1 was 783.9 ± 46.7 for non-stimulated cultures of BxPC-3 and TRAIL-treatment significantly increased the expression of DcR1 reaching a mean fluorescence intensity of 1083.0 ± 52.7 (*n* = 7, *p* = 0.0009). Regarding the expression of DcR2 in BxPC-3 cell cultures, the mean fluorescence intensity was 287.4 ± 24.5. This was significantly increased following TRAIL-stimulation to a mean fluorescence intensity of 522.3 ± 40.9 (*n* = 8, *p* = 0.0002, Fig. 4a).

Co-stimulation with TRAIL and LPS significantly further decreased the expression of DR4 and further increased the expression of DcR2 by BxPC-3 cell cultures: The expression of DR4 by BxPC-3 cell cultures decreased from 722.4 ± 86.0 in TRAIL-stimulated cell cultures to 440.0 ± 81.6 in cell cultures co-stimulated with LPS and TRAIL (*n* = 6, *p* = 0.03, Fig. 4a).

Regarding the expression of DcR2 by BxPC-3 cell cultures, the mean fluorescence raised from 522.3 ± 40.9 in TRAIL-stimulated cell cultures to 631.8 ± 69.0 following co-stimulation with TRAIL and LPS (*n* = 8, *p* = 0.1). However, this effect did not reach significance.

Co-stimulation with LPS and TRAIL did not significantly influence the expression of neither DR5 nor DcR1 when compared to TRAIL-stimulated cell cultures. Regarding the expression of DR5 by BxPC-3 cells, the mean fluorescence intensity was 1017.5 + − 22.0 in TRAIL-stimulated cell cultures. In co-stimulated cell cultures the mean fluorescence intensity was 861.4 ± 112.3 (*p* = 0.2, *n* = 8). Regarding the expression of DcR1, the mean fluorescence intensity was 1083.43 ± 52.7 (*n* = 7) in TRAIL-stimulated cells and 1001.0 ± 75.7 in co-stimulated cell cultures of BxPC-3 (*n* = 8) (*p* = 0.4; Fig. 4a).

### Pancreatic cancer cell lines expressed TLR4 and TRAIL-stimulation decreased the expression of TLR4

**Fig. 5:**
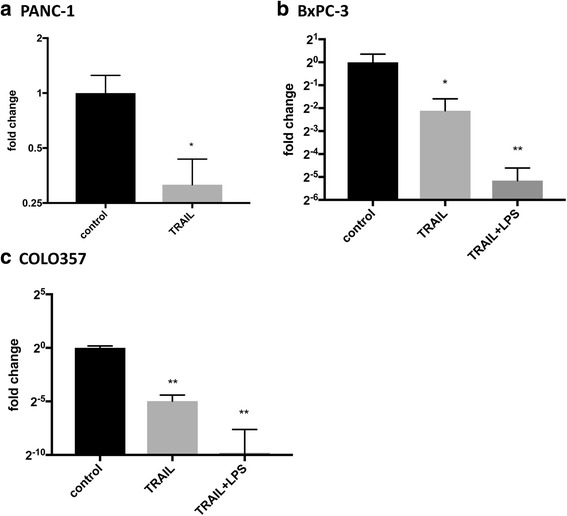
TRAIL-stimulation decreased the mRNA-levels of TLR4. Cultures of PANC-1 (**a**), BxPC-3 (**b**) and COLO357 (**c**) were stimulated with 300 ng/ml TRAIL and 300 ng/ml TRAIL + 1 μg/ml LPS for 24 h. Non-stimulated cell cultures served as controls. TLR4 mRNA levels were measured by quantitative realtime-PCR. Data were normalized to mRNA expression of a housekeeping gene, *GAPDH*, and shown as fold change compared to untreated cells. Means and standard errors of the mean are shown. *) *p* < 0.05; **) *p* < 0.01

As TLR4 is the receptor for LPS, we investigated the expression of this receptor in pancreatic cancer cell lines. As shown by RT-PCR, all of the investigated pancreatic cancer cell lines expressed TLR4. Yet, TRAIL-stimulation decreased the expression of TLR4 in all investigated cell lines: TRAIL-stimulation of PANC-1 cell cultures led to a 3.2-fold decrease in the expression of TLR4 (Fig. 5a). In BxPC-3 cells, TRAIL-stimulation decreased the TLR4 expression 4.4-fold (Fig. 5b). Co-stimulation with LPS and TRAIL further decreased the expression of TLR4 resulting in a 35.3-fold decrease when compared to non-stimulated cultures of BxPC-3 cells (Fig. 5b). In COLO 357 cells, TRAIL-stimulation induced a 31.1-fold decrease in the expression of TLR4. Co-stimulation with TRAIL and LPS further decreased TLR4 reaching a 933.3 fold decrease when compared to non-stimulated COLO 357 cell cultures (*n* = 5/group, Fig. 5c).

## Discussion

In this study, it could be shown that LPS-stimulation inhibited TRAIL-induced apoptosis in pancreatic cancer cell lines by modulating the expression patterns of respective TRAIL-receptors.

TRAIL is a member of the TNF superfamily and was initially thought to selectively induce apoptosis in cancer cells [28]. However, more recently it has been discovered that TRAIL also exerts a negative impact on immune cells [27, 29, 30] demonstrating promotion of tumour growth in a murine model of pancreatic cancer [31].

To avoid excessive apoptosis induction following TRAIL-stimulation, many tumour cells developed several mechanisms to counteract TRAIL-induced apoptosis. Several mechanisms can involve different steps of the TRAIL signalling pathway. Firstly, NF-kB mediated survival mechanisms have been discovered involving genes like X-linked inhibitor of apoptosis (XIAP). Secondly, the expression pattern of different TRAIL-receptors can contribute to TRAIL-resistance. Whereas the expression of TRAIL-R1 (DR4) and TRAIL-R2 (DR5) contributes to TRAIL-induced apoptosis [32, 33], these effects can be counteracted through the binding of TRAIL to the decoy receptors DcR1, DcR2 and osteoprotegerin [34]. Variations in the expression of these receptors will thus influence the impact on TRAIL-induced apoptosis.

Previously it has been shown that sensitivity to TRAIL-induced apoptosis may differ between cell lines of pancreatic cancer cells: PANC-1 has been detected to be TRAIL-resistant, whereas the cell line BxPC-3 appears to be TRAIL-sensitive. This effect was contributed to KRAS mutations in PANC-1 cell lines [22, 35]. This present study confirmed the finding: COLO 357 displayed a high sensitivity to TRAIL-induced apoptosis, BxPC-3 displayed an intermediate sensitivity and PANC-1 cells were resistant to TRAIL-induced apoptosis.

Previous results could confirme the expression of the TRAIL-receptors DR4, DR5, DcR1, DcR2 and osteoprotegerin showing that these receptors were expressed by all investigated cell lines [22]. Additionally, it could be shown that all investigated cell lines secreted osteoprotegerin (OPG). However, there were significant differences between the investigated cell lines regarding the regulation of the expression of TRAIL-receptors following TRAIL-stimulation. In the TRAIL-resistant cell line PANC-1, TRAIL-stimulation significantly decreased the expression of the death receptors DR4 and DR5 whereas the secretion of osteoprotegerin was significantly increased. The levels of OPG in PANC-1 cell cultures were highest, confirming reports linking K-RAS mutations in pancreatic cancer cells with levels of osteoprotegerin [36]. Remarkably, in the present study TRAIL-stimulation increased OPG-levels thereby decreasing the expression of death receptors. Levels of OPG were lowest in the KRAS wild type cell line BxPC-3. TRAIL-stimulation had no influence on OPG secretion. However, the expression of DR4 was decreased following TRAIL-stimulation and the expression of the decoy receptors DcR1 and DcR2 was increased resulting in an intermediate resistant phenotype. In contrast, the cell line COLO 357 displayed a high sensitivity to TRAIL-mediated apoptosis. Following TRAIL-treatment the levels of OPG were decreased whereas the expression pattern of the receptors DR4, DR5, DcR1 and DcR2 did not alter.

Toll-like receptors are a major class of pattern recognition receptors which recognize highly conserved microbial structures called pathogen-associated molecular patterns (PAMPs) allowing the immune system to identify a variety of pathogens [7]. Therefore, TLRs are able to detect pathogens and subsequently initiate an immediate immune response.

TLR4 which binds LPS was the first Toll-like receptor to be identified. Therefore, it plays an important role in innate immunity. Thus, the activation of TLR4 by bacterial LPS mounts a pro-inflammatory reaction yielding in the elimination of the pathogen [37].

Apart from their expression on immune cells, Toll-like receptors have been identified in many other cell types including endothelial cells, myocytes and thyreocytes. Additionally, TLR expression has been found on pancreatic beta-cells, alpha-cells and even ductal cells [38].

In pancreatic ductal adenocarcinoma an increased expression of TLR4 has been detected compared to adjacent normal tissue [39]. In this study, we have confirmed and expanded these investigations showing that all investigated pancreatic cancer cell lines expressed Toll-like receptor 4.

TLR4 appears to act as a double-edged sword as it has been linked to both cancer inhibition and growth [40]. Whereas TLR4−/− mice showed decreased tumour growth in a murine model of pancreatic cancer, inhibition of MyD88 accelerated tumour development [41] as well as an increased TLR4 expression correlated with tumour size, lymph node involvement, venous invasion and pathological stage [39]. Most reports regarding pancreatic cancer and TLRs however focus on interactions of respective ligands with immune cells of the tumour’s microenvironment. With lung cancer cells it has been shown that TLR4-signalling promoted resistance towards TRAIL-induced apoptosis and that this effect could be induced by LPS-stimulation [26]. For the pancreatic cancer cell lines PANC-1 and AsPC-1 it has been demonstrated that LPS-stimulation increased the invasive ability of respective cell lines through NFkB signalling [5].

This study showed an effect of LPS-signalling on the sensitivity to TRAIL-induced apoptosis in pancreatic cancer cell lines. This effect has been detected in all investigated TRAIL-sensitive cell lines and showed a strong reduction of TRAIL-induced apoptosis. In BxPC-3 cells, this effect was accompanied by an increase in the expression of DcR1 and a decrease of DR4-expression whereas the expression of DR5 and DcR2 remained unchanged when compared to TRAIL-stimulation. There were no changes detected in the secretion of OPG. In contrast, in COLO 357 cells the expression of the TRAIL-receptors was not changed comparing TRAIL-stimulated cell cultures with cell cultures stimulated with TRAIL and LPS. However, there was a significant increase in the level of osteoprotegerin contributing to TRAIL-resistance following co-stimulation [36].

Despite this fundamental role of LPS on TRAIL-function, the significance of TLR4 in this context has yet to be analysed. All cell lines expressed TLR4 but its level of expression was significantly reduced by TRAIL-stimulation. Therefore, only small numbers of functional TLR4s are required for LPS-mediated TRAIL-inhibition.

In summary, TLR4 was expressed on all analysed pancreatic cancer cell lines. LPS-stimulation decreased TRAIL-induced apoptosis. The decreased resistance to TRAIL-induced apoptosis was accompanied by alteration in the patterns expression of TRAIL-receptors. Future TRAIL-therapeutic strategies must be aimed at restoring TRAIL sensitivity by increasing functional TRAIL-receptors, blocking decoy receptors and other TRAIL-binding proteins as well as by counter-acting the inflammatory micro-milieu in pancreatic cancer. The role of LPS and TLR4 has to be further elucidated in future studies.

## Conclusions

In this study, it has been shown that TRAIL-resistance in pancreatic cancer cells can be mediated by the inflammatory molecule LPS as well as by different expression patterns of functional and non-functional TRAIL-receptors.

## Abbreviations

DR: Death receptor
ELISA: Enzyme-linked immunosorbent assay
LPS: Lipopolysaccharide
MFI: Mean fluorescence intensity
OPG: Osteoprotegerin
PCR: Polymerase chain reaction
TLR: Toll-like receptor
TRAIL: TNF-related apoptosis inducing ligand
TRAIL-R: TRAIL-receptor
